# Viral Infection and Lung Cancer Immunotherapy

**DOI:** 10.3389/fonc.2021.577514

**Published:** 2021-08-09

**Authors:** Ewa Kalinka, Izabela Chmielewska, Kamila Wojas-Krawczyk

**Affiliations:** ^1^Department of Oncology, Polish Mother’s Memorial Hospital – Research Institute, Lodz, Poland; ^2^Department of Pneumonology, Oncology and Allergology, Medical University of Lublin, Lublin, Poland

**Keywords:** lung cancer, immunotherapy, HBV, HCV, HIV, SARS-Cov-2

## Abstract

Immunotherapy with immune checkpoint inhibitors (mainly anti-PD1 and anti-PDL1 monoclonal antibodies) became a standard of care in non-small cell lung cancer (NSCLC) patients. Most of the clinical trials excluded patients with hepatitis B (HBV), hepatis C (HCV), and human immunodeficiency virus (HIV) active infection (1–10). Despite the progress in treatment of these infections, they remain an unresolved clinical problem when lung cancer immunotherapy should be initiated in an NSCLC patient. This manuscript summarizes the data from the literature concerning this subgroup of patients including the rationale for immunotherapy initiation depending on the HBV, HCV, or HIV infection status; the risk of adverse events; and the efficacy compared to non-infected patients. One of the crucial questions is how the candidates to immunotherapy should be screened for HBV, HCV, and HIV infections. The year 2020 brought the world a new but dynamic viral problem—severe acute respiratory syndrome coronavirus 2 (SARS-Cov-2). The incorporation of known data in oncology guidelines became a burning need, and then, which group of the infected patients can be treated with immunotherapy despite the infection. Oncologists should also know if these patients should receive antiviral therapy and what are the safe combinations in these settings. We also indicate which of the adverse events should be monitored carefully during checkpoint inhibitor treatment.

## Introduction

The landscape of advanced non-small cell lung cancer (NSCLC) with no epidermal growth factor receptor (EGFR) or anaplastic lymphoma kinase (ALK) genomic tumor aberrations treatment has evolved substantially during the past few years (1–10). Immunotherapy was a promising potential intervention in lung cancer. The cancer immunoediting phenomenon comprises the following stages: elimination, equilibrium, and escape. In the elimination stage, immunosurveillance proper priming and effector phase of the host is efficient enough to obtain tumor elimination. In the equilibrium phase, the immune system allows the malignancy presence but the host can still control it, avoiding cancer progression. In the escape phase, the immune system passively allows proliferation and tumor growth without active control mechanism (1). Thus, the ideal therapeutic intervention would transform the immune escape to elimination phase. Therapeutic strategies that allow achievement of equilibrium phase are not curative, but possibly lead to overall survival (OS) improvement despite the lack of cancer elimination. As NSCLC cells are moderately immunogenic, equilibrium is an achievable goal for immune checkpoint inhibitors and allows malignancy control in an important proportion of patients (11).

The incorporation of immune checkpoint inhibitors (ICIs) started when nivolumab showed superiority over docetaxel in second-line treatment in the CA 209-017 and CA 209-057 trials (3, 4). Then, atezolizumab showed OS benefit in second-line treatment over docetaxel in the OAK and POPLAR trials. These trials established ICI as the standard of care in NSCLC patients progressing after platinum-doublet first-line treatment (3–6).

Then, KEYNOTE trials showed that pembrolizumab gives further benefit in first-line treatment in NSCLC patients. First, pembrolizumab in monotherapy showed survival benefit over platinum doublet chemotherapy in treatment-naive patients with programmed death receptor type 1 (PD-L1) expression of at least 50% in the KEYNOTE-024 trial (7). KEYNOTE-407 and KEYNOTE-189 trials demonstrated that the combination of pembrolizumab with platinum doublet chemotherapy led to OS benefit compared to chemotherapy alone in first-line treatment of NSCLC patients with untreated NSCLC, regardless of the PD-L1 status and the histological subtype (8, 9). Thus, treatment-naïve advanced NSCLC patients were treated with pembrolizumab monotherapy if PD-L1 expression was 50% or higher from 2016 or immunochemotherapy in any case from 2018.

The year 2020 brought a new Food and Drug Administration (FDA) registration based on CA 209-227 trial—nivolumab combined with ipilimumab was found superior to chemotherapy alone in an untreated NSCLC patient in whom PD-L1 expression was ≥1%. The most common adverse events (AEs) observed in ≥20% of patients receiving the combination of nivolumab plus ipilimumab, as in other studies using this combination, were the following: (1) general: fatigue, rash, decreased appetite, and musculoskeletal pain; (2) gastrointestinal: diarrhea/colitis, nausea, and hepatitis; (3) respiratory: dyspnea and cough; and (4) dermatological: pruritus (10).

In January 2021, the results of the CheckMate 209-9LA trial were published, showing that in untreated NSCLC patients, nivolumab plus ipilimumab combined with two cycles of platinum-based chemotherapy improve OS versus standard arm. The study demonstrated that addition of ICI therapy shortens chemotherapy duration with no unexpected safety signs (12). During the past months, this trial’s results were incorporated into recommendations modifications in this clinical setting.

In the vast majority of trials evaluating ICI in NSCLC patients, the infection with hepatitis B (HBV), hepatis C (HCV), or human immunodeficiency virus (HIV) was an exclusion criterion and none of them included patients with SARS-Cov-2 (3–6, 10, 12). Thus, we cannot drive our clinical decisions on evidence-based medicine as literature does not provide data on scientific evidence of good quality level.

On the other hand, HCV-, HBV-, and HIV-infected patients are at increased risk of developing malignancies, and this population should not be omitted if the most effective treatment option is based on ICI. Precise knowledge of potential risk and specific approach is necessary if such a therapy is to be initiated.

Since 2020, the oncologists have to face the problem of SARS-Cov-2 infection among lung cancer patients. A basic knowledge about clinical presentation, susceptibility, and outcome are substantial to take proper clinical decisions.

This manuscript provides a literature review in these special populations of NSCLC patients treated with ICI. The presented manuscripts are summarized in **Table 1**.

**Table 1:** Selected manuscripts presenting data concerning ICI therapy in HCV/HBV/HIV infected patients.

Study design	Patient population	Number of patients	Result	First author
Case series	NSCLC and MM with HCV/HBV infection	7	1 patient with ALT G2 elevation improved after anti-HCV treatment initiation, the remaining 6 with no clinically significant hepatic toxicity	Kothapalli A (13)
Retrospective 5 centers analysis	Advanced stage cancers with HCV/HBV/HIV infection	50	HIV–irAE rate 24%, grade ≥3 14%, no changes in viral load or CD4+ count HCV/HBV–irAE rate 44%, grade ≥3 29%—no reactivation observed	Shah NJ (14)
Systematic review	Advanced cancers with HIV infection	73	In 6 out of 70 patients, grade ≥3 irAE occurred In 26 out of 28 patients, viral load remained undetectable CD4+ lymphocytes count increased where data are available Response rates 30% for NSCLC, 27% for MM and 63% for KS	Cook MR (15)
Retrospective study	NSCLC and HCV/HBV infection	19	No hepatic irAE requiring ICI discontinuation Overall response rate—35%	Pertejo-Fernandez A (16)
Retrospective prospective single-institution study	HCC and HBV infection	62	No HBV reactivation in NUC-treated patients In reactivation out of six patients without NUC prophylaxis	Lee PC (17)
Open-label phase 1	Pembrolizumab in HIV infection	33	No unexpected irAE Overall response rate showing feasible efficacy	Uldrick TS (18)
Retrospective cohort study	ICI in HBsAg-positive patients	114	HBV reactivation in 5.3% of patients HBV DNA monitoring and antiviral prophylaxis recommended	Zhang X (19)
Systematic review	Advanced cancer with HBV/HCV	89 HBV 98 HCV		Pu D (18)
Review	HBV reactivation preemptive approach in ICI-treated patients		HBV infection screening in all patients initiating ICI. Antiviral prophylaxis recommended in patients at risk of reactivation	Hwang JP (20)

ICI, immune checkpoint inhibitors; HCV, hepatitis C virus; HBV, hepatitis B virus; HIV, human immunodeficiency virus; NSCLC, non-small cell lung cancer; MM, malignant melanoma; KS, Kaposi sarcoma; HCC, hepatocellular carcinoma; ALT, alanine aminotransferase; NUC, nucleos(t)ide analogs.

## Human Immunodeficiency and Hepatitis B and C Virus

As previously mentioned, the infection with HIV, HBV, or HCV is one of the most common exclusion criterion for clinical trials incorporating ICI in the treatment plan.

In patients infected with HBV and HCV treated with ICI, the most common concern is potential HBV reactivation and immune hepatitis induced by ICI. Patients infected with HIV are additionally at risk of infectious complications of cancer therapy. Our experience with this patient population started based on case reports, but every year brings new larger evidence in these groups of patients.

In one of the published series, seven patients treated with PD-1 inhibitors nivolumab and pembrolizumab for either metastatic melanoma or metastatic NSCLC with medical history of chronic or past HBV/HCV infection treatment were analyzed retrospectively. One patient showed an increase in alanine transaminase (ALT) grade 2 severity that returned to the normal range after treatment of his HCV infection. In four other patients, ALT elevation grade 1 was noted, with no intervention needed, and in the two remaining patients, no toxicity was seen. Treatment outcome was similar as in the noninfected population. Based on these observations, the authors conclude that patients with NSCLC and metastatic melanoma can be effectively and safely treated with PD-1 inhibitors despite HBV/HCV infection, but a close cooperation with hepatologist is required if eventual antiviral therapy is indicated (13).

In a retrospective analysis from five centers, Shah et al. summarized the treatment course in 50 patients with advanced stage cancer and HBV, HCV, or HIV infection treated with ICI. In the HIV cohort of 21 patients, the rate of immune-related adverse events (irAE) was 24% with 14% if grade ≥3 irAE and no significant changes in viral load and CD4+ T-cell counts. In the HCV/HBV cohort of 34 patients, irAE rate was 44% with 29% grade ≥3. In the HBV/HCV patients with pre- or post-treatment viral load, reactivation was not observed (14).

In 2019, a systematic review aimed to assess the efficacy and safety profile of ICI therapy of advanced cancer patients with coexisting HIV infection. The authors identified 73 patients from 11 case reports and 2 case series. In this group, 62 patients were treated with anti-PD-1 agents, 6 with anti-CTLA-4, 4 with the combination of both anti-PD-1/anti-CTLA-4, and 1 with sequential ipilimumab and nivolumab. In only 6 of 70 patients were irAE of grade ≥3 observed, with good tolerance among others. In 26 of 28 patients with undetectable viral load pre- and posttreatment, their HIV remained undetectable. Importantly, CD4 T-cell counts increased in patients where data were available. Reported objective response rates were 30% for NSCLC, 27% for melanoma, and 63% for Kaposi sarcoma. Based on the presented results, ICI treatment in HIV-, HBV-, or HCV-infected patients was safe and effective with no new toxicity data (15, 16).

In order to assess pembrolizumab safety and efficacy in HIV-infected patients with a CD4+ count of at least 100 cells/µl and treated with antiretroviral therapy (ART) for at least 4 weeks, an open-label, phase 1 study was conducted in 33 patients (6 with Kaposi sarcoma, 5 with non-Hodgkin lymphoma, and the remaining 19 with non-AIDS-related cancers). In the cohort, 12 irAE occurred, namely, hypothyroidism (6), pneumonitis (3), rash (2), and aminotransferase elevation (1)—thus typical toxicity profile. All patients were on ART, and uncontrolled HIV infection was not noted in any subject. CD 4 T-cell counts increased during the study, but this finding was not statistically significant. The obtained response rates confirm that pembrolizumab is an effective and feasible therapy in HIV-positive cancer patients (18).

Important analysis results were recently published by Lee et al. (17) The retrospective prospective, single-institution study aimed to “review and follow-up consecutive 62 patients with chronic hepatitis B or resolved HBV infection who had received ICIs” for hepatocellular carcinoma (HCC)—thus a population that is high risk for hepatic irAE due to frequent underlying liver disfunction. No HBV reactivation occurred in the 35 patients with HBV DNA ≤100 IU/ml on nucleos(t)ide analog therapy (NUC) as well as in 19 patients in whom NUC was initiated with the ICI treatment. In six patients receiving ICI with no NUC protection, one developed HBV reactivation, while in three a greater than 1 log decrease in HBV viral load was observed. These data are similar to those in the CheckMate-040 trial, where 3 of 51 HBV-HCC patients (6%) presented a 1 log decline of HBV surface antigen (HBsAg) (17). Moreover, in patients receiving NUC, hepatic irAE occurred at the same level of about 10% of patients irrespective of viral load level. This small, but important real-life study provides evidence that ICI therapy can be safely administered even with detectable viral load, but as none of the patients experienced HBV reactivation while on NUC prophylaxis, this strategy during ICI therapy is strongly recommended.

Another retrospective cohort study enrolled 114 HBsAg-positive cancer patients treated with ICIs. HBV reactivation was diagnosed in 5.3% of patients with an undetected HBV DNA at baseline. HBV reactivation prophylaxis was given in only one of these six patients. None of the events was fatal. The authors found that the lack of antiviral prophylaxis was the only significant risk factor of HBV reactivation (OR 17.50, *p* = 0.004). The authors concluded that HBsAg-positive patients are at risk of HBV reactivation during ICI therapy and should be monitored for HBV DNA and given reactivation prophylaxis (19).

A few months ago, we could see the results of a retrospective study designed to assess the safety and efficacy of ICI in patients with history of HBV or HCV infection and NSCLC. The study population consisted of 19 patients with NSCLC and history of past or chronic HBV (16 cases; 2 of these had HCV co-infection) or chronic HCV infection (5 cases), who received an anti-PD-1 monoclonal antibody. An overall response rate was 35%. The liver function test elevation from baseline was mild and quite rare with no irAE of grade 3–4 requiring ICI discontinuation or steroids to treat hepatic toxicity. No cases of HBV reactivation or HCV flare were observed on ICI therapy, and no changes in viral load occurred. Thus, treatment of NSCLC patients with ICI appears quite safe in the population of HCV- or/and HBV-infected patients with efficacy measures consistent with those in the noninfected population of NSCLC patients (16).

A large systematic review was published in 2020 by Pu et al. It included data from 8 case reports, 4 case series, and 2 trials with 89 patients infected with HBV and 98 with HCV infection who received ICI (ipilimumab, nivolumab, pembrolizumab, atezolizumab, durvalumab, avelumab, and tremelimumab) for advanced stage cancer.

There were no treatment-related deaths, but some hepatic events occurred: In 3.4% of HBV-infected patients, and 17.3% of HCV-infected patients, grade 3 or 4 transaminase elevation was seen. Moreover, 2.8% without antiviral treatment experienced a virus load increase, and in 1.9%, virus-related hepatitis was diagnosed. In 18.6% with hepatocellular cancer, 32.4% with melanoma, and 16.7% of NSCLC patients, objective response was achieved. The authors conclude that ICIs are safe and effective in advanced cancer patients with HBV or HCV infection, but still reactivation of viral hepatitis are possible in an unclear mechanism. They recommend to initiate and continue antiviral therapy during ICI treatment if indicated (21).

An important review was published in 2021 with a recommendation to screen all the patients before ICI administration for serologic tests of HBV infection, including HbsAg, anti-Hbc, total IgG, and anti-HBs in order to identify all patients at risk of HBV reactivation to whom antiviral prophylaxis is strongly advised (20). This recommendation involves changes in practice and requires efforts in the field of oncologists’ education. It was shown that even in non-Hodgkin lymphoma and chronic lymphocytic leukemia treated with anti CD20 monoclonal antibodies (a high-risk population for HBV reactivation), the adherence to appropriate HBV screening guidelines is not sufficient, which shows the need of intensification of educational strategies in the global oncohematologic medical community (22).

Further important data for HIV-infected patients with malignancies were provided in the phase 2, open label, non-randomized DURAVAST trial. The trial included 20 patients with malignancies in which ICI therapy was approved or data proving anti-PD1 or anti-PD-L1 activity were published, with no available standard therapy at study enrollment. All the patients were on combination antiretroviral therapy and had no detectable viral load. The CD4+ and CD8+ lymphocyte count and viral load were monitored during durvalumab therapy (at a dose of 1500 mg every 28 days), and no significant changes in these HIV infection activity parameters were observed. Moreover, the study showed a disease control rate of 50%, with 25% of partial responses with no new safety signals in this specific population. The authors conclude that, if indicated, the durvalumab monotherapy should be available to patients with controlled HIV infection (23).

To summarize, HBV, HCV, and HIV infection in NSCLC patients can be treated with ICI if indicated in most of the cases (24, 25). As evidence-based data from prospective clinical trials are still lacking in these populations, ICI therapy initiation requires viral load baseline assessment and close monitoring of specific hepatic irAE in HCV/HBV-infected patients and additionally CD4+ T cell count in HIV patients. Moreover, in treatment planning, oncologists should take into account potential interactions of ICI and antiviral therapy if needed. The occurrence of irAE is especially dangerous in HIV patients in whom immunosuppressive drugs could deteriorate the infection control with increased risk of opportunistic infections. That is why a closer monitoring of irAE occurrence is needed in this patient group.

In the authors’ opinion, the modification of HBV/HCV/HIV-infected patients requiring ICI therapy should include:

a. HBV-infected patients
- Reactivation prophylaxis in every patient at risk at least 7 days before ICI initiation
- AST, ALT, and total bilirubin concentration assessment before each ICI administration—if any of them increases, viral load should be monitored
- If reactivation is diagnosed, ICI therapy should be delayed until liver tests return to normal and ICI rechallenge should be discussed with hepatologist
b. HCV-infected patients
- Anti-HCV therapy initiation should be considered in every case, with assessment of viral load at day 15. If decreased, ICI can be safely initiated (17, 21). If anti-HCV treatment is not effective at day 15, ICI therapy is still possible, but safety should be discussed with a hepatologist
- AST, ALT, and total bilirubin concentration assessment before each ICI administration—if any of them increases, viral load should be monitored
- If reactivation is diagnosed, ICI therapy should be delayed until liver tests return to normal and ICI rechallenge should be discussed with a hepatologist
c. HIV-infected patients
- Every patient should receive anti-HIV treatment
- CD4+ lymphocyte count and viral load should be assessed before ICI initiation
- Monitoring of CD4+ lymphocyte count and viral load should be monitored by an infectious disease specialist as in standard practice
- Organ-specific anti-HIV drug toxicity should be monitored before each ICI administration in order to avoid cumulation of toxicity mechanisms

According to published data, there is no need to assess ICI efficacy more frequently than in the rest of the population.

## SARS-Cov-2 Virus

The landscape of lung cancer immunotherapy AEs and clinical course of SARS-Cov-2 infection in lung cancer patients has substantially influenced oncologists’ approach to treatment since January 2020. Theoretically, immunotherapy in lung cancer patients could potentially enhance immunologic control and improve COVID-19 and other infection outcome, but it could also lead to increase the risk of COVID-19 complications, especially in the hyperactive immune phase.

Since the pandemic started in China, the first reports originated from Wuhan. In March 2020, a group of Chinese researchers published a retrospective analysis of three hospitals’ database in Wuhan; clinical data were collected from medical records from patients with confirmed COVID-19 (26).

The experience from 14 hospitals included 105 cancer patients (20.95% with lung cancer) and a matched control group, all with COVID-19. The authors demonstrated higher rates of death, intensive care unit (ICU) admission, having at least one severe or critical symptom, and higher risk of the need of mechanical ventilation in the cancer patients’ group, especially in the metastatic setting. It was observed that patients who received immunotherapy tended to have high rates of death [two (33.33%) of six patients] and high chances of developing critical symptoms [four (66.67%) of six patients]—the numbers were very small and a significant difference could not be shown (27).

The data from the systematic review of 31 studies show 181,323 patients with COVID-19, of whom 23,736 patients with cancer confirmed that they are at increased risk of mortality and morbidity. The mortality was highest in hematological malignancies (OR 2.43) followed by lung cancer (OR 1.8) with no association between a particular type of oncologic therapy (28).

Luo et al. were the first authors to address the question whether PD-1 blockade therapy affects COVID-19 severity in lung cancer patients. In the analyzed population, 41 (59%) patients previously received PD-1 blockade in four categories: ever received PD-1 blockade, most recent dose within 6 months, most recent dose within 6 weeks, and first dose within 3 months. Overall, there was no significant difference in severity regardless of PD-1 blockade exposure. Peak IL-6 level among hospitalized patients was similar based on receipt of PD-1 blockade. In this single-institution cohort, over half of our patients with lung cancers and COVID-19 required hospitalization and almost a quarter died, which is consistent with findings from Hubei province (27, 29).

One of the latest published reports used a global database (TERAVOLT) in order to establish what are the effects of SARS-Cov-2 infection in thoracic malignancies patients (26). Data were obtained from 200 patients (151 with NSCLC) from eight countries; most of the patients were in active treatment at the time of SARS-Cov-2 infection. As in the general population, the most common presenting symptoms were fever, dyspnea, and cough with the most frequent complications being pneumonia or pneumonitis and acute respiratory distress syndrome. Again, as previously reported in the first Chinese observations (27), a high mortality rate of 33% was confirmed in this patient population. Despite the fact that 76% of the chest malignancy population required hospitalization, there was a low rate of admission to ICUs of 9% with mechanical ventilation for 6% of patients only. Of the 66 patients who died, 79% of deaths were attributed to COVID-19 complications only and only 1 (2%) to complications to cancer therapy. Although univariate analysis showed that age above 65 years, active or past smoking history, treatment with chemotherapy alone, and the presence of comorbidities were associated with higher risk of death, in the multivariate analysis, only smoking history was associated with increased risk of death during SARS-Cov-2 infection in thoracic malignancy patients (3.18; 1.11–9.06). The study found no evidence that the type of systemic therapy affected survival; nevertheless, patients treated with TKIs were at decreased risk for hospitalization (30).

The most recent manuscript was dedicated to identify the patient-specific and cancer-specific features that impact severity of COVID-19, as clinical decisions should be driven by this knowledge. The study was based on a single-institution experience, the Memorial Sloan Kettering Cancer Center in New York, and included 102 consecutive lung cancer patients with COVID-19 diagnosis and analyzed the severity of COVID-19 outcome. The authors found that COVID-19 in lung cancer patients is associated with severe clinical course, with 62% requiring hospitalization and a 25% rate of fatal outcome. As in the general population, patient-specific risk factors including smoking status and chronic obstructive pulmonary disease (odds ratios for severe COVID-19 2.9, 95% CI 1.07–9.44 comparing the median [23.5 pack-years] to never and 3.87, 95% CI 1.35–9.68, respectively) correlated with severe outcome (hospitalization, ICU stay, intubation and invasive mechanical ventilation intubation, and/or transition to do not intubate or death). What is encouraging in taking decisions to refer lung cancer patients to ICUs is the fact that 25% of patients initially requiring intubation recovered from COVID-19, as well as the majority of the studied population. The examined impact of cancer therapy on the COVID-19 clinical course and outcome remains a critical question that should drive real-life clinical decisions during the pandemic. In the studied population, recent PD-1 blockade with or without chemotherapy was not linked with increased severity of COVID-19 as well as the comparison of chemotherapy or TKI. The authors did not find an improvement in severity of clinical course of hydroxychloroquine in the studied population (31).

A single-institution retrospective analysis included 820 cancer patients in whom SARS-Cov-2 infection was diagnosed. The observed rates of respiratory failure or death were 36% and 32% for metastatic lung cancer patients who did and did not receive immunotherapy, respectively. The authors admit that immunotherapy use, thoracic cancer, smoking history, and metastatic solid cancer are highly associated; it is difficult to understand which factors are responsible for worse COVID-19 outcomes and need larger analyses in cancer-specific cohorts (32).

The prospective observational study provided more optimistic evidence—the study included 800 patients with active cancer and confirmed SARS-Cov-2 infection by RT-PCR. The clinical course was mild in 52% of patients. Although a high mortality rate of 28% was observed, the risk of death was associated with age (odds ratio [OR] 9.42, *p* < 0.001), male gender (OR 1.67, *p* = 0.003) and presence of comorbidities with an important role of hypertension (OR 1.95, *p* < 0.001) and cardiovascular disease (OR 2.32) but not with chemotherapy (even administered up to 4 weeks before COVID-19 diagnosis), immunotherapy, radiotherapy, hormone, or targeted therapy. Thus, the impact of cancer diagnosis and/or treatment itself on COVID-19 clinical course and especially the risk of death was not confirmed (33). A small prospective study was designed in Italy to evaluate the incidence and clinical course of COVID-19 in 53 cancer patients treated with ICI. It was observed that influenza-like illness occurred in 8 of them, but only 3 with lung cancer had a confirmed diagnosis of SARS-Cov-2 infection. The low-resolution computed tomography revealed interstitial pneumonia in all 3 cases with 30%, 50%, and 40% of affected lung volume, respectively. All these patients were hospitalized with respiratory failure diagnosis, and two of them died due to acute respiratory distress syndrome—elderly males with cardiovascular comorbidities; the third patient recovered from COVID-19 (34). This study shows that once COVID-19 in ICI-treated cancer patients has a severe course, it is associated with respiratory complications and high mortality, especially if additional risk factors such as age, gender, and comorbidities increase the risk.

As more data were published in 2019 and 2020, a meta-analysis including 3,581 cancer patients with COVID-19 could be planned. The authors found that infected patients who recently received anti-cancer treatment (including surgery, chemotherapy, targeted therapy, immunotherapy, and radiotherapy) were not at higher risk of COVID-19 exacerbation or death. The cancer treatment-related factors with a negative impact on COVID-19 clinical course were as follows: (1) chemotherapy administered within 28 days before infection—associated with increased risk of death (OR 1.45, *p* = 0.015, *p* = 0.015 for interaction) and (2) immunotherapy associated with the risk of exacerbation (OR 2.53, *p* = 0.006, *p* = 0.170 for interaction) (35).

Thus, as reported above, we have different signals concerning the impact of chemotherapy or immunotherapy on COVID-19 endpoints, and in each case, decisions have to be made in a personalized manner in non-vaccinated patients. As the COVID-19 pandemic is still ongoing, clinicians have to consider the potential risk in patients initiating ICI therapy. Important expert opinions support ICI initiation in a metastatic setting in patients without COVID-19. The adjuvant setting prolonging progression-free survival only in ICI therapy is not equally justified in the authors’ opinion due to increased risk of healthcare resources—an important risk factor of SARS-Cov-2 infection transmission. Treatment delay can also be advised in low volume indolent malignancy (36). We do not have enough published data to predict COVID-19 clinical course in vaccinated patients; thus, special attention should be made before clear recommendations are issued.

Mid-2020 supported the oncological community with the first recommendations concerning lung cancer diagnosis and treatment in the COVID-19 era. Authors are consistent and bring a similar approach to advanced-stage NSCLC patients. It is clearly emphasized that oncologists “should still follow the principles of providing the best possible care and palliative management of our patients to improve overall survival and maintain quality of life”. As we all agree that it is expected to reduce the oncology centers’ visit frequency, patients can benefit from trials results that have shown that less frequent anti-PD-1 antibody administration does not influence their efficacy and safety (37, 38). These data allow to prolong intervals between immunotherapy administration and decrease the number of visits in a medical institution, which is one of the most important measures in terms of risk of SARS-Cov-2 infection.

COVID-19 brought a new challenge for oncologists—the differential diagnosis of COVID-19 and immune-related adverse events in the course of immunotherapy. As shown in the literature, both immune-related pneumonitis and SARS-Cov-2-related pneumonia have similar clinical manifestations, with the most frequent symptoms occurring in both populations being cough, dyspnea, fever, hypoxia, and weakness. Moreover, the occurrence of gastrointestinal symptoms is also likely a symptom of both COVID-19 and irAE in the course of ICI treatment (39–41). Radiological differences between these two etiologies do not give enough tools to make a certain differential diagnosis (39, 41). The obvious minimal diagnostic panel must include SARS-Cov-2 infection test and its result defines further treatment. Thus, if the infection is confirmed, the patient should be treated as having COVID-19, but if it is negative, the therapy should cover immune-related pneumonitis without any delay. The exclusion of SARS-Cov-2 infection should be made based on approved testing methods, i.e., molecular testing with or without antigen test in justified cases. It is important to avoid the synergy in drug and infection lung injury mechanisms, which could be life threatening, especially in NSCLC patients who have multiple additional risk factors for adverse pneumonia outcome. It is also advised to check SARS-Cov-2 status before ICI rechallenge after the toxicity is resolved for the abovementioned reasons.

Since the late 2020, anti-SARS-Cov-2 vaccines have become available. As a sufficient number of doses and healthcare resources were not sufficient, governments all over the world had to establish which populations should be vaccinated as a priority. Thus, the populations in which COVID-19 has an unfavorable clinical course compared to the general population had to be determined. Based on important literature review, it was confirmed that cancer patients should be vaccinated as early as possible due to the high risk of COVID-19 complications and mortality, especially higher in patients with hematological malignancies and lung cancer in advanced stages or those undergoing oncological treatment as frequent contact with healthcare workers is an additional risk factor for SARS-Cov-2 infection (42).

In May 2021, the first results showing the safety of the two doses of the BNT162b2 mRNA vaccine in 134 ICI-treated cancer patients were compared to the control group. Similar rates of side effects were noted in both groups (pain at the injection site, muscle pain, fatigue, headache, fever, chills, and gastrointestinal or flu-like symptoms). The study did not show new irAE or exacerbation of the previously noted side effects. Moreover, in patients with irAE, the vaccine side effects were not more frequent and had a mild clinical presentation (43). These data seemed encouraging as available literature for influenza vaccine showed higher incidence of grade 3 or 4 irAE in vaccinated patients receiving nivolumab or pembrolizumab compared to patients who were not vaccinated. In addition, some series reported an increased influenza syndrome rate in vaccinated patients undergoing ICI treatment compared to unvaccinated subjects. Published studies show that ICI efficacy is not impaired in influenza vaccinated patients; even better cancer control results were reported in the literature (44). We should not extrapolate results obtained for influenza vaccines to those obtained for the BNT162b2 mRNA vaccine as the first one is based on attenuated viruses while the anti-SARS-Cov-2 vaccines are based on a different technology, which results probably in a different pattern of efficacy and irAE occurence. Lately, we were provided evidence that patients with solid tumors undergoing COVID-19 vaccination demonstrate a high anti-spike immunoglobulin G antibody (IgG-Ab) positivity of 98% and 97% in patients treated with ICI. It was observed that mRNA-based vaccines are associated with higher IgG-Ab titers than adenoviral ones (45).

As SARS-Cov-2 infection is a new phenomenon, it is strongly advised to follow updated recommendations available, for example, on American Society of Clinical Oncology or European Society of Medical Oncology websites (46, 47).

To summarize the data we have at the end of 2020, lung cancer patients are more susceptible to SARS-Cov-2 infection and they are at higher risk of developing COVID-19 complications such as pneumonia/pneumonitis and ARDS, of being admitted to the ICU, of using mechanical ventilation, and, unfortunately in about one-third of cases, of dying. We do not have clear indications that patients undergoing ICI, subjected to systemic therapy, are at a different risk of contracting COVID-19 or have a different prognosis than patients treated with other systemic therapies. In 2021, when SARS-Cov-2 vaccines are becoming more and more accessible for cancer patients, it is strongly advised to perform the vaccination in order to avoid COVID-19 in cancer patients. In the non-vaccinated patients, national or local policies concerning SARS-Cov-2 molecular screening before immunotherapy initiation differ substantially between countries and even institutions. Despite that, it seems justified to screen every lung cancer patient for SARS-Cov-2 infection before therapy initiation and every subsequent dose, especially in the non-vaccinated population. If the patient is SARS-Cov-2-positive (even if asymptomatic), the delay of any cancer therapy initiation until full recovery (at least 2 weeks following resolution of symptoms) is the only known reasonable approach (36). The question how often testing should be repeated remains open. Also, testing of family members or people living within the same home is a reasonable option as many healthy adults and children can be infected but asymptomatic with high risk of transmission to the lung cancer patient in whom the clinical course can be severe or even fatal.

## Author Contributions

EK was responsible for literature search and review, publications choice, data assembly, proper language corrections, manuscript writing and final approval. IC was responsible for manuscript writing, proper language corrections and final approval. KW-K was responsible for literature search and review, publications choice, data assembly, proper language corrections, manuscript writing and final approval. All authors contributed to the article and approved the submitted version.

## Conflict of Interest

EK: honoraria from Roche, Merck, Sharp and Dohme, Bristol-Myers Squibb, AstraZeneca. KW-K: honoraria from Roche, Merck, Sharp and Dohme, Bristol-Myers Squibb, AstraZeneca.

The remaining author declares that the research was conducted in the absence of any commercial or financial relationships that could be construed as a potential conflict of interest.

## Publisher’s Note

All claims expressed in this article are solely those of the authors and do not necessarily represent those of their affiliated organizations, or those of the publisher, the editors and the reviewers. Any product that may be evaluated in this article, or claim that may be made by its manufacturer, is not guaranteed or endorsed by the publisher.
